# Retraction: Aqueous extract of Bambusae Caulis in Taeniam inhibits PMA-induced tumor cell invasion and pulmonary metastasis: Suppression of NF-κB activation through ROS signaling

**DOI:** 10.1371/journal.pone.0355253

**Published:** 2026-08-04

**Authors:** The PLOS One Editors

Following the publication of this article [1], concerns were raised with results presented in Figs 4 -7 and the statistical analysis reported in the article. Specifically,

- The following panels appear to partially overlap:
  - Lanes 3-4 and lanes 9-10 of the Fig 4A pIκBα panel and lanes 1-2 and lanes 3-4 of the Fig 6B pIκBα panel, respectively.
  - The second Migration panel of Fig 7B and the first Invasion panel of Fig 7B.
  - The third and the fourth Migration panels of Fig 7B.
- There appear to be irregularities suggestive of splice lines in the following panels:
  - Fig 5 t-ERK
  - Fig 7D IκBα
- The y-axis of the middle and the bottom graphs of Fig 5 bear the same label, although the graphs represent different measurements.
- The Materials and Methods section only reports the use of Student’s t-test, and does not explain how the analysis was adjusted for multivariate comparisons or corrected for multiple pairwise comparisons.

First author AK responded stating that the original data underlying the panels of concern are no longer available due to the age of the article. Regarding the partial panel overlap concerns, first author AK acknowledged that the indicated regions appear to partially overlap in the published figures but stated that, in the absence of the original data, they could not verify the origin of the panels or exclude the possibility of inadvertent figure assembly errors. They also stated that the apparent irregularity may have been introduced during figure assembly when the two condition blocks shown in the Fig 5 t-ERK panel were arranged for direct comparison. First author AK clarified that the bottom graph was mislabelled and should read p-JNK/JNK. In the absence of the original blot and image data, the image concerns with Figs 4-7 cannot be fully resolved.

Regarding the statistical concerns, first author AK stated that no multivariate statistical analysis or multivariate modelling was performed in this study. They stated that the statistical annotations referred to comparisons of individual experimental endpoints between the relevant control and treatment groups and that no formal adjustment for multiple pairwise comparisons was applied. PLOS remains concerned about the statistical approach used in this article.

In light of the above unresolved concerns, which call into question the reliability and integrity of the published results, the *PLOS One* Editors retract this article.

AK and NHY did not agree with the retraction. MJ, YPJ, and JYM either did not respond directly or could not be reached.
